# Human gene editing: revisiting Canadian policy

**DOI:** 10.1038/s41536-017-0007-2

**Published:** 2017-01-05

**Authors:** Bartha Maria Knoppers, Rosario Isasi, Timothy Caulfield, Erika Kleiderman, Patrick Bedford, Judy Illes, Ubaka Ogbogu, Vardit Ravitsky, Michael Rudnicki

**Affiliations:** 1 Centre of Genomics and Policy, McGill University, Montreal, QC Canada; 2 Department of Human Genetics, Institute for Bioethics and Health Policy, John P. Hussman Institute for Human Genomics, Interdisciplinary Stem Cell Institute, Miami, FL USA; 3 Health Law Institute, University of Alberta, Edmonton, AB Canada; 4 Clinical Translation and Regulatory Affairs, Centre for Commercialisation of Regenerative Medicine (CCRM), Toronto, ON Canada; 5 National Core for Neuroethics, Division of Neurology, Department of Medicine, University of British Columbia, Vancouver, BC Canada; 6 Faculty of Law and Faculty of Pharmacy and Pharmaceutical Sciences, University of Alberta, Edmonton, AB Canada; 7 Bioethics Program, Department of Social and Preventive Medicine, School of Public Health, University of Montreal, Montreal, QC Canada; 8 Regenerative Medicine Program, Ottawa Hospital Research Institute, Ottawa, ON Canada; 9 Department of Cellular and Molecular Medicine, Faculty of Medicine, University of Ottawa, Ottawa, ON Canada

## Preamble

Driven by the rapid evolution of gene editing technologies, international policy is examining which regulatory models can address the ensuing scientific, socio-ethical and legal challenges for regenerative and personalised medicine.^1^ Emerging gene editing technologies, including the CRISPR/Cas9 2015 scientific breakthrough,^2^ are powerful, relatively inexpensive, accurate, and broadly accessible research tools.^3^ Moreover, they are being utilised throughout the world in a wide range of research initiatives with a clear eye on potential clinical applications. Considering the implications of human gene editing for selection, modification and enhancement, it is time to re-examine policy in Canada relevant to these important advances in the history of medicine and science, and the legislative and regulatory frameworks that govern them. Given the potential human reproductive applications of these technologies, careful consideration of these possibilities, as well as ethical and regulatory scrutiny must be a priority.^4^

With the advent of human embryonic stem cell research in 1978, the birth of Dolly (the cloned sheep) in 1996 and the Raelian cloning hoax in 2003, the environment surrounding the enactment of Canada’s 2004 Assisted Human Reproduction Act (AHRA) was the result of a decade of polarised debate,^5^ fuelled by dystopian and utopian visions for future applications. Rightly or not, this led to the AHRA prohibition on a wide range of activities, including the creation of embryos (s. 5(1)(b)) or chimeras (s. 5(1)(i)) for research and in vitro and in vivo germ line alterations (s. 5(1)(f)). Sanctions range from a fine (up to $500,000) to imprisonment (up to 10 years) (s. 60 AHRA).

In Canada, the criminal ban on gene editing appears clear, the Act states that “No person shall knowingly […] alter the genome of a cell of a human being or in vitro embryo such that the alteration is capable of being transmitted to descendants;” (s. 5(1)(f) AHRA). This approach is not shared worldwide as other countries such as the United Kingdom, take a more regulatory approach to gene editing research.^1^ Indeed, as noted by the Law Reform Commission of Canada in 1982, criminal law should be ‘an instrument of last resort’ used solely for “conduct which is culpable, seriously harmful, and generally conceived of as deserving of punishment”.^6^ A criminal ban is a suboptimal policy tool for science as it is inflexible, stifles public debate, and hinders responsiveness to the evolving nature of science and societal attitudes.^7^ In contrast, a moratorium such as the self-imposed research moratorium on human germ line editing called for by scientists in December 2015^8^ can at least allow for a time limited pause. But like bans, they may offer the illusion of finality and safety while halting research required to move forward and validate innovation.

On October 1^st^, 2016, Health Canada issued a Notice of Intent to develop regulations under the AHRA but this effort is limited to safety and payment issues (i.e. gamete donation). Today, there is a need for Canada to revisit the laws and policies that address the ethical, legal and social implications of human gene editing. The goal of such a critical move in Canada’s scientific and legal history would be a discussion of the right of Canadians to benefit from the advancement of science and its applications as promulgated in article 27 of the Universal Declaration of Human Rights^9^ and article 15(b) of the International Covenant on Economic, Social and Cultural Rights,^10^ which Canada has signed and ratified. Such an approach would further ensure the freedom of scientific endeavour both as a principle of a liberal democracy and as a social good, while allowing Canada to be engaged with the international scientific community.

## Points to consider

We maintain that current Canadian policy and regulatory instruments appropriately address somatic cell research, including the gene editing of such cells. However, in light of new discoveries and capabilities, and evolving societal perceptions,^11^ a reconsideration of policies for germ line editing is warranted. We propose that:

1. There is a scientific and societal value in promoting research, including research that may involve germ line modification techniques in human embryos or gametes prior to the stage of implantation.
2. The modification of human germ cells in the context of non-clinical research should be allowed in Canada.
3. Criminal bans are not a suitable instrument to regulate scientific research. Justifications for upholding the current approach should be revisited and new objectives and mechanisms of future policy broached.
4. A principled and pragmatic approach should underlie any discussion about future advances in human gene editing, including possible transition to the clinical context.

## Policy instruments

While there may be other interpretations of section 5(1)(f) AHRA, we consider that allowing gene editing in the context of research, including pre-clinical research on germ cells prior to implantation (i.e., 14 days of development) (s. 5(1)(d) AHRA) conforms to the spirit of the Act. As a first step, a guidance document on the interpretation of section 5(1)(f) AHRA from Health Canada would enable a clear understanding of this section and the scope of the criminal sanctions contained therein. Moreover, further debate could culminate in the revision of the AHRA itself and section 5(1)(f) specifically, as well as provide regulatory guidance, and the empowerment of a current government entity or the creation of a new entity for oversight.

Revision of the AHRA is long overdue. Parliament specifically directed that the AHRA be reviewed 5 years from when it came into force. Human germ line editing can serve as the catalyst for such discussion and revision.
